# Dysphagia from Diastasis of the Cornua of the Os Hyoides

**Published:** 1830-01-01

**Authors:** 


### Difficulty of Swallowing from Dislocation or Diastasis of the Cornua of the Os Hyoides.

Sauvages in his nosology has given the name of the dysphagia of Valsalva, to the difficulty of swallowing arising from this cause, which was first described by the latter author. Since his time Borsieri and Molinelli have recorded several cases of the affection in question, sometimes occasioned by external violence, sometimes from swallowing large morsels of food. Dr. Gio. Bat. Magna has recently published a similar case in the Annali Universali di Medicina, of Milan, for November and December, 1828, of which we shall make a short abstract.

A man of sixty, extremely meagre and flabby, whilst endeavouring to swallow a large piece of the tendon of beef, thought he felt it stick in his throat, and made many attempts to get it down without being even able to swallow his saliva. Each unsuccessful effort was accompanied with a peculiar noise of air gurgling up the œsophagus, but respiration and the power of speech were free. Nothing could be seen nor felt about the pharynx or neck, and a bougie passed readily down the œsophagus, without encountering any obstacle or procuring any relief. The painful spot being precisely the region of the os hyoides, and no foreign body being lodged there, Signor Magna imagined that dislocation or rather diastasis of the appendices of the os hyoides itself, might be the cause of all the symptoms. He accordingly passed down behind the base of the tongue the fore and middle fingers of the right hand, and moved the os hyoides in the manner recommended by Valsalva and Sauvages, whilst the left hand was applied on the bone behind. Immediately the uneasy sensations experienced by the patient in the spot disappeared, and he was able to swallow water with ease.

Two years afterwards a similar accident happened from taking a large mouthful of hard. cold bouillie, and was remedied by similar means.

